# Artificial Intelligence in Tumor Evolution: Understanding Cancer Complexity Through Multi-Modal Data Integration in Precision Oncology

**DOI:** 10.3390/cells15111031

**Published:** 2026-06-03

**Authors:** Asunción Espinosa-Sánchez, Amancio Carnero

**Affiliations:** 1 Instituto de Biomedicina de Sevilla (IBIS), HUVR/CSIC/Universidad de Sevilla, 41013 Sevilla, Spain; aespinosa-ibis@us.es; 2 CIBER de Cancer (CIBERONC), Instituto de Salud Carlos III, 28029 Madrid, Spain

**Keywords:** evolutionary oncology, intratumoral heterogeneity, multi-omics integration, single-cell transcriptomics, spatial transcriptomics, deep learning, convolutional neural networks, tumor microenvironment, adaptive therapy, evolutionary game theory, clonal evolution, precision oncology

## Abstract

**Highlights:**

**What role does AI play in evolutionary oncology?**

- AI integrates multi-omics, imaging, and clinical data to model tumor evolution.
- Machine learning improves detection of heterogeneity and therapy resistance.

**How does AI support precision cancer treatment?**

- AI enables prediction of adaptive tumor trajectories and drug response.
- Evolutionary oncology frameworks support adaptive and precision therapies.

**Abstract:**

Cancer research has undergone a fundamental transformation in recent decades due to the integration of artificial intelligence (AI) models into the study of tumor biology. However, tumor evolution, driven by genetic and phenotypic alterations leading to heterogeneity, resistance and metastasis, remains a major challenge in oncology. To understand these processes is crucial for developing effective therapeutic strategies and improving patient outcomes. Conventional methods often fail to capture the complexity and dynamics of these processes. In contrast, AI tools have the ability to integrate and analyze large-scale multi-omics, imaging and clinical data, offering the capability to decode tumor complexity. AI-driven methods facilitate multi-modal data integration, enabling the recognition of patterns that connect molecular alterations with phenotypic outcomes. In functional genomics, AI tools predict the effects of genetic variants, identify regulatory elements and map dysregulated pathways, thus clarifying mechanisms underlying tumor development and resistance. In the imaging field, deep learning techniques improve tumor segmentation, characterization and longitudinal monitoring, providing more accurate insights into tumor progression and treatment response. Predictive modeling could allow the anticipation of tumor evolution and drug response, supporting adaptive therapeutic plans and real-time treatment adjustments. Moreover, AI supports biomarker discovery, patient stratification and decision support systems that can improve clinical trial design and accelerate the development of personalized therapies. However, these advances raise important ethical challenges, including data privacy, algorithmic bias and the preservation of patient autonomy. Addressing these concerns is essential to ensure the responsible deployment of AI in oncology.

## 1. Introduction

Tumor evolution is a central driver of cancer progression, metastasis, and therapeutic resistance. The dynamic accumulation of genetic, epigenetic, and phenotypic alterations generates intratumoral heterogeneity that challenges durable clinical control.

The exponential growth of high-throughput technologies—including whole-genome and whole-exome sequencing, single-cell RNA sequencing (scRNA-seq), spatial transcriptomics, chromatin accessibility profiling (ATAC-seq), and circulating tumor DNA (ctDNA) liquid biopsy platforms—has enabled the generation of massive, heterogeneous datasets spanning the genomic, transcriptomic, proteomic, epigenomic, and radiomic dimensions of cancer [1,2,3,4]. The depth and dimensionality of these data vastly exceed the analytical capacity of conventional statistical methods, creating an urgent need for computational frameworks capable of integrating and interpreting complex, multi-modal biological information at scale.

Artificial intelligence (AI) has emerged as a powerful approach to address these limitations. However, its role in tumor evolution research is often framed as a collection of analytical tools applied to isolated data modalities. AI has progressively filled this gap: machine learning (ML) algorithms excel at identifying genomic and clinical predictors of disease risk and treatment outcome, while deep learning (DL) architectures—convolutional neural networks (CNNs), recurrent neural networks (RNNs/LSTMs), graph neural networks (GNNs), variational autoencoders (VAEs), and transformer-based models—enable the extraction of hierarchical features from raw sequencing reads, histopathological images, and multi-omic matrices alike. Generative models, such as generative adversarial networks (GANs), address data scarcity through biologically informed data augmentation, while graph-based frameworks capture the relational architecture of gene regulatory and protein–protein interaction networks [1,5].

Recent advances have expanded AI applications across the full tumor evolution pipeline, from deep learning-based variant calling and single-cell atlas integration to transformer models for regulatory genomics, graph-based multi-omic stratification and fragmentomics-driven liquid biopsy [6,7,8]. The breadth and pace of these developments underscore the need for a systematic synthesis that places them within a unified evolutionary framework.

Despite this remarkable progress, several critical research gaps persist. First, the majority of AI models in oncology are trained and validated on single-institution, single-modality datasets, limiting their generalizability across sequencing platforms, tumor types and patient populations [9]. Second, most published models treat cancer as a static snapshot rather than a dynamic evolutionary process: they lack the capacity to integrate longitudinal multi-omic data in a temporally coherent manner, which is essential for tracking clonal sweeps, epistatic interactions, and the emergence of resistant subclones over time [10]. Third, the tumor microenvironment (TME)—a key modulator of evolutionary dynamics through immune editing, metabolic competition, and stromal remodeling—remains poorly represented in current predictive models, in part because its spatial and cellular complexity is only now accessible through spatial transcriptomics and highly multiplexed imaging [11]. Fourth, the interpretability of deep learning models remains a fundamental barrier to clinical translation: without mechanistic insight into model predictions, it is difficult to distinguish genuine biological signal from technical artifact or data leakage [12]. Fifth, prospective validation of AI-based adaptive therapy strategies in clinical trials is virtually absent, underscoring the gap between algorithmic innovation and bedside application [9].

In this review, we argue that AI is most transformative when conceptualized as an integrative evolutionary inference engine—one that enables reconstruction of clonal dynamics, alignment of heterogeneous longitudinal data, and anticipation of resistance trajectories through the coordinated analysis of multi-modal cancer datasets. Accordingly, this review is organized around the core evolutionary questions of cancer biology, rather than around data types or algorithms: (i) how intratumoral heterogeneity arises and persists, (ii) how tumors adapt under therapeutic pressure, (iii) how the tumor microenvironment shapes evolutionary trajectories, and (iv) whether future tumor states can be anticipated to guide adaptive clinical strategies. This perspective highlights both the opportunities and the limitations of AI in translating evolutionary insight into clinical benefit.

## 2. Conceptual Framework: AI as an Evolutionary Inference Engine

Tumor evolution unfolds across multiple interconnected biological layers. First, variation is generated through genetic mutation, chromosomal instability, epigenetic remodeling, and phenotypic plasticity. Second, selective pressures arising from microenvironmental constraints and therapeutic interventions shape clonal competition and cooperation. Third, tumors undergo temporal adaptation, with clonal architectures continuously reshaped over the course of disease progression and treatment.

Within this evolutionary framework, AI fulfills four interrelated roles. AI models enable the reconstruction of latent clonal states from noisy and incomplete data; align heterogeneous signals across genomic, transcriptomic, spatial, imaging, and clinical modalities; simulate evolutionary responses to therapeutic perturbations; and translate evolutionary predictions into clinically actionable decision support. This framework shifts emphasis from algorithmic catalogs toward biologically and clinically meaningful evolutionary inference.

## 3. The Complexity of Tumor Evolution

Tumor evolution is driven by the continuous interplay between genetic diversity, phenotypic plasticity, and environmental selection. Intratumoral heterogeneity emerges through the accumulation of point mutations, copy number alterations, and structural variants, coupled with non-genetic mechanisms such as transcriptional reprogramming and microenvironmental adaptation. These processes generate coexisting subclonal populations with distinct fitness landscapes, complicating therapeutic control.

AI-based analyses of bulk and single-cell sequencing data have improved resolution of clonal architectures and revealed latent subpopulations that may drive progression or resistance. Nevertheless, most current approaches rely on static snapshots and struggle to distinguish neutral drift from selection-driven clonal expansion, underscoring the need for temporally resolved and evolution-aware modeling strategies [13,14].

### 3.1. Genetic Heterogeneity

#### 3.1.1. Intratumor Heterogeneity

Intratumoral heterogeneity (ITH) refers to the coexistence of different genetic subpopulations within a tumor. This diversity originates from the accumulation of mutations, genomic instability, microenvironmental influences and the ability of the tumor to adapt to cancer therapies [15,16].

In relation to mutations, point mutations, insertions, deletions and copy number variations are included. In the case of driver mutations, they confer a selective growth advantage and contribute to cancer progression. However, passenger mutations are considered not to directly affect tumor behavior [17,18]. Moreover, tumors undergo a process of clonal expansion, causing those subpopulations with an advantage in proliferation to grow faster than others. This principle of Darwinian selection leads to a hierarchical organization inside the tumors, in which dominant clones emerge while numerous minor subpopulations coexist [14,19,20]. ITH has been reported in some types of tumors. For instance, in non-small cell lung cancer, different tumor regions may hold some EGFR or KRAS mutations, which complicates targeted therapy strategies [21]. In glioblastoma, the presence of extrachromosomal DNA carrying amplified oncogenes such as EGFR has been linked to rapid tumor evolution and therapeutic resistance [22]. In addition, in HER2-positive breast cancer, intratumoral heterogeneity in ERBB2 amplification correlates with poor response to HER2-targeted therapies [23].

Our current understanding of intratumoral heterogeneity is still limited. However, the advances in DNA sequencing technologies and the application of AI in cancer research could represent a significant step forward in this area.

#### 3.1.2. Mechanisms of Genetic Diversity

Several mechanisms contribute to the genetic diversity observed in tumors [24]. One of the main factors is genomic instability, a hallmark of cancer cells that leads to an increased mutation rate and promotes the accumulation of genetic alterations. This instability can result in defects in DNA repair mechanisms, such as mismatch repair, homologous recombination and non-homologous end joining [20]. In the case of colorectal and endometrial cancers, the appearance of defects in mismatch repair is frequent, leading to microsatellite instability [25,26]. However, BRCA1/2 mutations that impair homologous recombination are more characteristic of breast and ovarian cancer [27]. Another cause of genetic diversity is chromosomal instability, which plays a significant role in driving tumor heterogeneity. This mechanism is characterized by the gain and loss of whole chromosomes or large chromosomal segments, promoting the possible appearance of aneuploidy. The aneuploidy cells have an abnormal chromosome number and affect gene expression and cellular behavior [28]. For instance, chromosomal instability is often linked to poor prognosis and resistance to standard treatments in triple-negative breast cancer [29,30]. Beyond these alterations, epigenetic modifications also influence tumor diversity. Mechanisms such as DNA methylation, histone modification, and non-coding RNAs regulate gene activity without changing the DNA sequence itself. These reversible and dynamic alterations modulate genes involved in proliferation, differentiation and survival, promoting adaptability and progression [31,32]. In glioblastoma, the DNA methylation patterns of the MGMT promoter were detected, and this alteration was associated with a better response to temozolomide therapy [33]. Similarly, it occurred in acute myeloid leukemia, where mutations in epigenetic regulators such as DNMT3A or TET2 caused a chromatin alteration state and contributed to clonal expansion [34,35].

Cancer cells can become resistant to therapies through different genetic mechanisms. One common mechanism involves the acquisition of mutations that alter the target of treatment. In chronic myeloid leukemia (CML), mutations in the BCR-ABL gene can act upon tyrosine kinase inhibitors, such as imatinib, decreasing their efficacy [36,37,38,39,40]. Similarly, in non-small cell lung cancer (NSCLC), secondary mutations in the EGFR gene can block the binding with EGFR inhibitors, like gefitinib and erlotinib [41,42,43,44]. Furthermore, cancer cells may also acquire multidrug resistance (MDR) by overexpressing drug efflux transporters, such as P-glycoprotein (P-gp), which actively remove chemotherapeutic agents from the cell and diminish their impact upon them [45,46,47,48,49].

### 3.2. Phenotypic Plasticity

Phenotypic plasticity refers to the ability of cancer cells to adopt different phenotypes in response to environmental alterations and selective pressures. This adaptability is a hallmark of tumor evolution and plays a critical role in cancer progression and metastasis [50,51,52].

#### 3.2.1. Epithelial–Mesenchymal Transition (EMT)

One of the most studied processes of phenotypic plasticity is the epithelial–mesenchymal transition (EMT). EMT is the process in which epithelial cells lose their cell–cell adhesion properties and acquire a more migratory and invasive mesenchymal phenotype. This transition is essential for cancer cells to disseminate from the primary tumor and establish metastasis at distant sites [53,54,55,56]. The transcriptional regulation of EMT is triggered by some transcription factors such as SNAIL, SLUG, TWIST and ZEB. These transcription factors repress epithelial markers, such as E-cadherin, and induce mesenchymal markers, such as N-cadherin and vimentin [57,58]. Moreover, differentiated cancer cells can acquire stem cell-like properties in response to microenvironment signals. Hypoxia [59], cytokines, interaction with stromal cells [60] and other extracellular matrix components can induce EMT. In particular, transforming growth factor-beta (TGF-β) is a potent inducer of EMT and promotes the invasive behavior of cancer cells [61,62,63].

In turn, EMT is also a key contributor to therapeutic resistance in cancer, acting upon both chemotherapy and targeted therapies. Mesenchymal-like phenotype cells tend to be more resistant to apoptosis and usually show an increase in drug efflux capabilities. Approaches that target the EMT process or its associated mesenchymal markers are being explored as a way to overcome this resistance [64,65,66].

#### 3.2.2. Cancer Stem Cells (CSCs)

Cancer stem cells, or CSCs, represent another aspect of phenotypic plasticity [67]. CSCs are a subpopulation of cancer cells with self-renewal and pluripotency capabilities. Those characteristics, plus their potential to differentiate into various cell types within the tumor, confer an important role in tumor initiation, progression and recurrence due to their unique properties [52]. The maintenance and function of CSCs are tightly regulated by key signaling pathways such as Wnt, Notch and Hedgehog, among others. The aberrant activation of those pathways supports the survival of CSCs and contributes to their resistance to conventional treatments [68]. Understanding the molecular mechanisms explaining that phenotypic plasticity of CSCs is essential for improving the therapeutic management of metastasis, recurrence and resistance to therapies [68,69]. However, it is important not only to focus on targeting CSCs, but also to consider that differentiated cancer cells can revert and acquire a stem-like phenotype by a process called de-differentiation [70]. In a recent study, the reduction in PSMG2 led to a decrease in the de-differentiation process and stemness in head and neck cancer cells, possibly through proteasome inhibition [71].

Cancer stem cells (CSCs) further illustrate the role of plasticity in therapy resistance. Their efficient DNA repair mechanisms, ability to enter a quiescent state and the increased expression of drug efflux transporters give them a significant advantage to be less sensitive to conventional treatments [69]. Current research is focused on strategies that specifically target CSCs or inhibit the pathways that maintain their stemness, in order to reduce tumor recurrence [72,73,74].

#### 3.2.3. Tumor Microenvironment

The tumor microenvironment (TME) plays an important role in tumor evolution by creating unique conditions that drive changes in the genetic and phenotypic landscape of cancer cells. The TMEs are composed of various types of cells, including immune cells, cancer-associated fibroblasts, endothelial cells, pericytes and extracellular matrix components [75,76,77,78]. The composition and function of the TME are different according to the tumor type, the patient and even tumor stage, adapting according to the conditions to protect the tumor [77]. Thus, the TME is the main process responsible for tumor plasticity.

#### 3.2.4. Immune Evasion and Therapy Resistance

To evade immune response, cancer cells use several strategies allowing them to persist and expand within the host [79,80]. The upregulation of checkpoint proteins is one common mechanism. The binding of PD-L1 and PD-1 receptors on T cells inhibits their cell activity, so immune checkpoint inhibitors, such as anti-PD-1 and anti-PD-L1 antibodies, have shown promising results in restoring anti-tumor immunity [81,82,83]. Additionally, the TME is enriched with immunosuppressive cells, such as regulatory T cells (Tregs) [84], myeloid-derived suppressor cells (MDSCs) [85] and tumor-associated macrophages (TAMs) and cancer-associated fibroblasts (CAFs) [86]. These cells secrete cytokines and growth factors that suppress immune responses, promote tumor proliferation and resistance to therapies [87,88,89,90,91]. Besides these mechanisms, the TME can also block effective immunotherapy by excluding cytotoxic T cells or by creating an immunosuppressed environment in which T cells are present but dysfunctional due to their interactions with suppressive cell populations [92]. In this context, current research is testing combinations of checkpoint blockade with changes in the TME. Examples include the administration of immunostimulatory cytokines like IL-12 and IL-15 to activate NK cells [93], as well as the use of engineered IL-2 variants to enhance T cell function while limiting Treg activation [94]. Extracellular matrix components, like collagen and fibronectin, could create a barrier to drug penetration and facilitate cell signaling pathways that promote survival and resistance [95,96,97].

#### 3.2.5. Angiogenesis

Tumor growth and metastasis depend on the formation of new blood vessels, a process known as angiogenesis. The TME provides pro-angiogenic signals that stimulate the proliferation and migration of endothelial cells to form new capillaries [80,98]. In the tumor microenvironment, hypoxia plays an important role in driving tumor plasticity. Hypoxia triggers hypoxia-inducible factors (HIFs), which promote the production of several angiogenic factors, including VEGF (Vascular Endothelial Growth Factor). This process not only supports the development of new blood vessels but also contributes to a more aggressive phenotype of tumor cell populations [61,99]. Moreover, tumor cells frequently have an upregulation in VEGF, which interacts with their receptors on endothelial cells and promotes their survival, growth and migration [100,101].

### 3.3. Evolutionary Dynamics and Tumor Progression

Understanding the evolutionary dynamics of tumors is essential to develop novel, effective therapeutic strategies. Tumor progression is a result of the interplay between genetic mutations, phenotypic plasticity and selective pressures from the TME and therapeutic interventions.

#### 3.3.1. Adaptive Evolution

Tumor cells continuously adapt to their microenvironment through a process of adaptive evolution. Various selective processes, including hypoxia, nutrient deprivation and immune surveillance, drive the emergence and expansion of subclones with advantageous adaptive [102]. Moreover, therapeutic interventions can also drive genetic selection, allowing only those subclones that possess resistance mechanisms to survive and continue to proliferate and invade [103]. These resistant subclones may accumulate additional mutations that enhance their capabilities and contribute to tumor progression. As a result, a periodic clonal sweep could occur, in which a subclone with a selective advantage quickly expands and dominates the tumor cell population. Moreover, these clones can reshape both the genetic and phenotypic characteristics of the tumor [102,103,104].

#### 3.3.2. Metastatic Evolution

Metastasis refers to the process by which cancer cells can spread into distant sites, one of the main causes of cancer-related mortality. This expansion mechanism has a series of coordinated and complex steps, including local invasion, intravasation into blood or lymphatic vessels, transport in the vessels, extravasation into distant tissues and a successful colonization [105]. The seed and soil hypothesis determines that metastasis is not a random event. In this case, some cancer cells (the “seeds”) are characterized as possessing intrinsic properties that allow them to invade and colonize specific organs (the “soil”) [106]. To achieve this, metastatic cells undergo a variety of genetic and epigenetic alterations that provide advantageous characteristics for migration and invasion processes, such as the upregulation of proteolytic enzymes and the activation of pro-survival and motility-associated pathways [107].

### 3.4. Strategies to Address Tumor Evolution

Given the complexity of tumor evolution, effective cancer treatment requires strategies that address the genetic and phenotypic diversity of tumors, as well as their dynamic interactions with the TME.

#### 3.4.1. Combination Therapies

One effective approach involves combination therapies that simultaneously target multiple oncogenic pathways. For instance, the therapies combined could prevent the emergence of resistant subclones and improve overall treatment efficacy and the development of resistance.

The identification of synergistic effects in the combinations of drugs could significantly improve therapeutic outcomes. Several approaches are used to uncover such interactions, including both in silico analyses and in vitro assays. High-throughput screening methods and computational models play a key role in predicting and selecting the most effective drug pairings. Recent studies show how computational modeling and high-throughput screening can complement each other for identifying drug synergies. DECREASE study demonstrated that machine learning could accurately predict drug synergies, significantly reducing in vitro assays [108]. Another study called MultiComb used deep learning to capture both drug response and synergy, improving the prediction reliability [109]. In myeloma, an experimental screening by high-throughput assays observed that certain drug combinations not only suppressed tumor growth but also affected key signaling pathways [110]. Following these strategies, a large-scale study in pancreatic cancer combined machine learning with experimental validation to uncover hundreds of effective combinations, showing the translational potential of this integrative approach [111].

Another promising strategy is sequential therapy, where different drugs are administered in a specific order. Studies demonstrated that the use of targeted therapy combinations significantly influences the evolution of drug resistance in a heterogeneous cancer cell population. This study suggests that in vitro long-term treatments could cause an increase in resistance mechanisms and could be a more realistic scenario of in vivo and clinical situations [112]. Additionally, in non-small cell lung cancer, a combined mathematical modeling and experimental assays were used to identify treatments that inhibit the growth of primary T790M-mutant cells and highlight the importance of sequential treatments in overcoming resistance to tyrosine kinase inhibitors [113]. This approach aims to exploit the evolutionary dynamics of tumor cells to prevent or delay the appearance of drug resistance.

Several clinical studies have demonstrated the effectiveness of combination therapies in overcoming resistance and improving patient outcomes. For instance, the combination of BRAF inhibitor vemurafenib with MEK inhibitor cobimetinib has significantly improved progression-free survival in patients with BRAF-mutant melanoma by targeting multiple nodes in the MAPK pathway simultaneously [114,115]. In lung cancer, the ongoing FLAIR trial (NCT04988607) is evaluating osimertinib plus bevacizumab versus osimertinib alone in treatment-naïve patients with EGFR exon 21 L858R-mutant NSCLC, aiming to improve outcomes in this less responsive subgroup [116]. Furthermore, the combination of immune checkpoint inhibitors, such as anti-PD-1 (nivolumab) with anti-CTLA-4 (ipilimumab), has demonstrated superior clinical benefit compared to monotherapy in various cancers, including melanoma and renal cell carcinoma [117]. These examples underscore the potential of rational drug combinations to exploit vulnerabilities in cancer signaling networks and delay the emergence of resistant clones.

#### 3.4.2. Adaptive Therapy

Adaptive therapy is an evolutionary treatment approach in which drug dosing is adjusted dynamically based on tumor response. Unlike conventional therapies that aim for the complete eradication of the tumor, adaptive therapy focuses on the maintenance of a stable population of drug-sensitive cancer cells. This strategy allows the competitive interactions between sensitive and resistant cancer cells, which, in turn, suppress the expansion of the resistant subclones population. Adaptive therapy is based on concepts like competitive release and the trade-offs in evolutionary advantages. To implement successful adaptive therapy, it could be necessary to have real-time monitoring of tumor dynamics. Techniques such as advanced imaging, liquid biopsy and molecular profiling can facilitate timely treatment adjustments [118,119].

Recent studies have demonstrated the feasibility of adaptive therapy in various cancers, including prostate and ovarian cancers, showing promising results in delaying tumor progression and reducing drug usage [120,121]. For instance, a pilot trial in metastatic castration-resistant prostate cancer achieved a median time-to-progression increase of over 10 months and a 53% reduction in cumulative drug usage compared to standard treatments [122].

#### 3.4.3. Targeting the Tumor Microenvironment

Another critical approach to managing tumor evolution involves targeting the tumor microenvironment (TME), which plays a central role in tumor progression and therapeutic resistance. One approach focuses on inhibiting pro-angiogenic factors, causing the deprivation of oxygen and nutrients in the tumor, thereby slowing its growth. Anti-VEGF agents, such as bevacizumab, have demonstrated clinical benefits in several cancers [123]. Another complementary strategy is to modulate the immune system. Therapies such as immune checkpoint inhibitors, cytokine treatments and adoptive cell transfer are being actively explored and have shown promising results across multiple malignancies [124].

## 4. Evolutionary Questions Addressed by AI

How does intratumoral heterogeneity arise and persist? AI approaches applied to bulk, single-cell, and spatial omics data have enabled the identification of latent cellular states and subclonal populations underlying tumor heterogeneity. Variational autoencoders, graph-based models, and clustering methods uncover structure obscured by technical noise and sampling bias. However, limited longitudinal sampling and assumptions of population stationarity constrain the ability of these models to fully reconstruct evolutionary trajectories [2,125,126].

How do tumors adapt under therapeutic pressure? Machine learning models integrating longitudinal genomics, circulating tumor DNA, and imaging data have demonstrated potential for early detection of resistance-associated changes. Despite these advances, many models remain correlational and lack explicit representations of evolutionary fitness landscapes. As a result, predictive performance often degrades when models are extrapolated across therapies or tumor contexts [126,127].

How does the tumor microenvironment shape evolutionary trajectories? AI-driven integration of spatial transcriptomics, imaging, and immune profiling has revealed critical non-cell-autonomous drivers of tumor evolution, including immune exclusion and stromal-mediated resistance. However, causal inference remains challenging, as most models inadequately capture bidirectional feedback between tumor cells and the microenvironment [125,128].

Can future tumor evolution be anticipated to guide therapy? Generative modeling and reinforcement learning approaches offer promising strategies to simulate tumor evolution under alternative treatment schedules, supporting adaptive and combination therapies. Clinical translation remains limited by data sparsity, simplified biological assumptions, and a lack of prospective validation [126,127].

## 5. AI in Genomic Data Analysis

**Figure 1:**
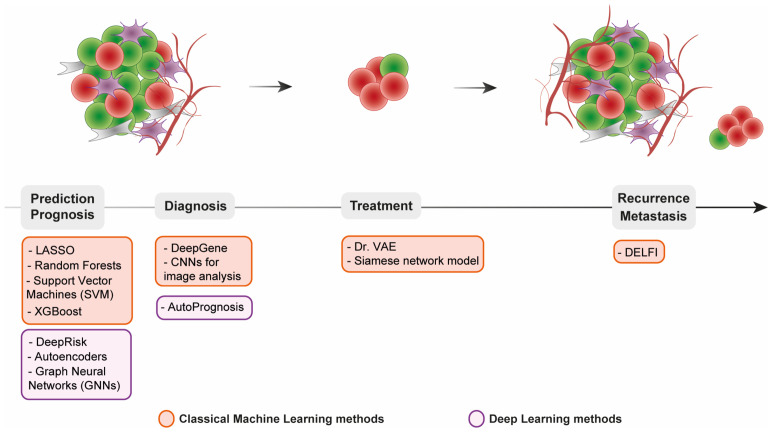
Machine learning and Deep learning techniques are used across the different steps of tumor evolution in AI-driven genomic data analysis. Orange: Classical ML methods; Purple: DL methods.

Artificial intelligence (AI) is transforming the field of genomic data analysis, providing powerful tools to decode the complexity of genetic information that drives some biological processes and diseases. In genomics, AI facilitates the interpretation of enormous datasets generated by next-generation sequencing (NGS), allowing researchers to detect genetic variations, understand molecular mechanisms and identify potential therapeutic targets. In particular, in the context of tumor evolution, these capabilities become especially valuable [129,130]. By combining AI-driven analysis with our understanding of how cancer adapts and develops resistance, we can better manage clonal dynamics, anticipate evolutionary evolution and design treatment strategies, such as adaptive or sequential therapies, that stay one step ahead of tumor progression [131,132]. This study explores the key roles of AI in genomic data analysis, highlighting its impact on data processing, pattern recognition and predictive modeling (Figure 1).

### 5.1. Data Processing and Management

Genomic data analysis begins with the processing and management of large-scale sequencing data. NGS technologies produce massive amounts of raw data, requiring efficient computational methods to process and store this information. AI, particularly machine learning (ML) algorithms, plays a crucial role in managing this data [129,130].

#### 5.1.1. Quality Control

AI algorithms are employed to perform quality control tests on sequencing data. These algorithms identify and filter out low-quality reads, correct sequencing errors and ensure that the data is accurate and reliable. Conventional tools like FastQC and MultiQC provide comprehensive reports on data quality, while Trimmomatic helps remove low-quality sequences and adaptors [133,134]. However, AI has allowed for the automation of these processes, reducing the time and effort required for manual quality assessment and improving both accuracy and efficiency. Tools like seqQscorer employ tree-based and deep learning models to evaluate features extracted from raw data and accurately predict which datasets are of low quality [135]. Another quality control AI tool was MAC-ErrorReads, which converts the search for erroneous reads into a supervised classification problem [136]. This tool achieves robust accuracy across some datasets using algorithms such as Naive Bayes. These AI-driven approaches not only reduce human workload but also enhance the precision and consistency of quality assessments, setting a new standard for efficiency in genomic preprocessing (Table 1).

#### 5.1.2. Data Compression

The storage and management of genomic data is a significant challenge due to the massive volume that it occupies. AI-based data compression techniques can efficiently encode genomic sequences, helping to reduce the storage requirements without compromising data integrity. They have developed models that combine convolutional layers with attention-based bidirectional LSTMs (Long Short-Term Memory), which use a neural network that looks at the sequence in both directions and focuses on relevant regions. This model can predict the probability of each nucleotide and use this information within an arithmetic encoder [137]. This approach achieved up to 3.7 times higher compression efficiency compared to existing methods like DeepDNA. In turn, DeepDNA itself applies a combination of CNN (Convolutional Neural Network) and LSTM architecture to compress human mitochondrial genomes [138]. GeCo3 (Genomic Compressor) is another specialized tool designed to efficiently capture the repetitive and structured nature of DNA, improving both reference-free and reference-based compression [139] (Table 1).

#### 5.1.3. Data Augmentation

Generative Adversarial Networks (GANs) are a deep learning method for data augmentation. Several state-of-the-art GAN architectures have been successfully applied in genomics and cancer research, demonstrating their ability to generate biologically plausible synthetic data. For example, ctGAN integrates gene expression and survival data to augment bulk RNA-seq datasets in cancer. In a recent study, ctGAN improved survival analysis performance across multiple cancer types by generating synthetic transcriptomic profiles that reflect both expression and patient outcome [5].

At the single-cell level, cscGAN has been used to realistically simulate scRNA-seq profiles by learning complex gene–gene dependencies, thereby improving downstream analyses such as marker-gene detection and classifier robustness. Moreover, scMMGAN extends this principle to multi-modal single-cell data: in a triple-negative breast cancer model, it integrates spatial RNA-seq and scRNA-seq modalities using a GAN framework with an added diffusion-geometry regularization term, producing coherent joint representations that respect the intrinsic geometry of each data modality [1].

In addition, more recent methods such as scMASKGAN (masked multi-scale CNN + attention-enhanced GAN) have been developed to impute scRNA-seq dropout events, improving the recovery of missing expression values [140]. Another example is LSH-GAN, which uses locality-sensitive hashing to accelerate sample generation for small-sample, high-dimensional scRNA-seq datasets; this has shown improvements in feature selection and clustering downstream [141] (Table 1).

#### 5.1.4. Pattern Recognition and Extraction

One of the most powerful applications of AI in genomics is its ability to recognize patterns and extract meaningful characteristics from complex data. These processes allow AI models to identify, quantify and interpret complex biological and clinical data that may be imperceptible to the human eye. In the oncology field, the integration of these techniques has significantly advanced the capacity to detect malignancies, predict outcomes and personalize the therapeutic strategy.

#### 5.1.5. Variant Calling

Variant calling is the process of identifying genetic variations, such as single nucleotide polymorphisms (SNPs) and insertions/deletions (indels), from sequencing data. AI algorithms, particularly deep learning models, have been developed to improve the accuracy and efficiency of variant calling. These models can analyze sequencing reads, detect variants with high precision and distinguish between true variants and sequencing errors.

Convolutional Neural Networks (CNNs) have been successfully employed for variant calling identification by transforming sequencing data into image-like representations such as pileup plots, in which the network learns to distinguish the real variant from sequencing noise or artifacts. The models aim to capture local spatial dependencies in read alignments, demonstrating a competitive performance compared to traditional identification methods based on rules or statistics, in particular, in managing low-frequency mutations or noisy sequencing regions. In oncology, CNNs can also extract latent features from raw or preprocessed sequencing data to correlate with specific mutational signatures or instability genomic patterns, which are characteristics of different cancer subtypes. These automated processes help to identify complex genomic variants and improve the sensitivity and specificity in the detection of somatic mutations. The direct learning from large- scale annotated cancer genome datasets has allowed models based on CNNs to generalize on different sequencing platforms and tumor subtypes, offering a potent tool to discover frequent oncology variants accurately and with high performance. Tools such as DeepVariant, DeepSom, NeuSomatic, VarNet and DeNovoCNN use CNNs to identify genetic variants by turning sequencing data into image-like representations. What differentiates them is their focus and input data. DeepVariant mainly focuses on germline mutations, particularly effective at detecting single nucleotide polymorphisms (SNPs) and small insertions or deletions (indels) in sequencing data from healthy individuals or patients [142]. In contrast, DeepSom and NeuSomatic are designed to detect somatic mutations, making them especially useful for cancer studies [7,143]. VarNet works with paired tumor and normal samples from the same patient to identify somatic mutations in the tumor [144], and DeNovoCNN aims to find de novo mutations, mainly applied in family-based studies to identify spontaneous genetic changes [6]. Together, these AI-based tools make variant detection more accurate, especially for low-frequency mutations (Table 1).

Recurrent Neural Networks (RNNs), in particular Long Short-Term Memory (LSTM) and Gated Recurrent Unit (GRU) architectures, have shown the capacity to model the sequential and contextual nature of genomic data and, in turn, identify variant calling. In contrast to CNNs, which are optimized for spatial pattern recognition, RNNs are designed to capture dependencies across ordered sequences, making them especially useful for analyzing nucleotide patterns along DNA strands. These tools can process aligned sequencing reads as sequences of bases, base quality scores and mapping information, learning to identify sequence motifs and positional dependencies to indicate the presence of true variants. In genomic regions where the local context is critical for distinguishing true somatic mutations from sequencing or alignment errors, these tools become particularly valuable, such as in repetitive elements, homopolymers or areas with complex structural variation. Moreover, RNNs can be used to model temporal or positional relationships in longitudinal omics data, such as changes in gene expression or mutational load across the disease’s progression or treatment. These temporal model capabilities allow the interpretation of variants to be more dynamic and personalized, above all, in studies of tumor evolution and resistance mechanisms. Some studies have employed tools based on recurrent neural networks (RNNs) for genomic analysis and variant detection. EvoLSTM applies a bidirectional LSTM encoder–decoder model to obtain sequence context and flanking nucleotides, enhancing the model of mutational processes [145]. Lokatt combines residual layers with LSTMs to improve nanopore base calling accuracy, a main step that directly impacts downstream variant identification [146]. DAVI compares basic RNNs and LSTMs for single nucleotide variant detection, showing that long-range dependencies are critical for accurate calling [147] (Table 1).

Even though RNNs are less commonly used than CNNs in current variant calling pipelines, they are being integrated into hybrid architectures to combine spatial and sequential modeling. These methods are designed to bring the strengths of different neural network architectures, making somatic variant detection more accurate and reliable, even in cancer datasets that are highly diverse and affected by noise.

#### 5.1.6. Gene Expression Analysis

Techniques to extract gene expression patterns across different cancer types, stages of the tumor and treatments have a crucial role in determining differences in molecular pathways, clonal dynamics and therapeutic responses. AI has become a key tool for studying the complexity of transcriptomic data, which often involves tens of thousands of genes measured across hundreds or thousands of patients. The conventional approaches are based on statistical methods, such as differential expression analysis or principal component analysis (PCA), and provide useful summaries but could miss the non-linear relationships that drive tumor behavior. AI models, particularly unsupervised and representation learning techniques, are increasingly used to discover hidden structures in high-dimensional transcriptomic data. These models can learn latent patterns that capture co-expression relationships, gene modules or pathway-level activity, which may not be visible through conventional analyses.

For instance, the use of Variational autoencoders (VAEs) has been applied to pan-cancer transcriptomic data to uncover latent features that capture biological variation. VAEs can be trained on TCGA RNA-seq data and can separate ovarian cancer subtypes, extracting patterns associated with survival outcomes [148]. Moreover, this approach can integrate with multi-omics layers in ovarian cancer, linking latent dimensions to clinical prognosis [149], while other models such as Dr.VAE have improved the prediction of drug response by learning pre- and post-treatment states [150]. At the single-cell level, models such as single-cell variational inference and extensions that integrate multi-omics layers (totalVI) allow the extraction of robust latent patterns that reveal transcriptional alterations and pathways relevant to therapeutic resistance and tumor progression [149,151] (Table 1).

Another relevant tool involves network-based deep learning, which has emerged as a powerful tool to analyze gene expression by incorporating previous knowledge of gene-gene interactions. Methods like Graph Convolutional Networks (GCNs) have been applied in breast cancer to identify co-expression modules linked to endocrine resistance, highlighting how selective therapy could shape clonal adaptation [152]. In the same way, similarity network fusion (SNF) combines multiple types of molecular data into integrated networks, allowing the discovery of patient subgroups with distinct transcriptional and evolutionary characteristics, as shown in lung and pancreatic cancers [153].

AI has also advanced cross-omics integration in transcriptomics. Tools such as MOFA+ and totalVI allow the joint analysis of RNA expression, copy number alterations and methylation or protein abundance models, capturing variation across molecular levels [149,154]. These methods have been used to bulk and single-cell data to connect transcriptional states to genetic backgrounds and microenvironmental contexts, providing a landscape of tumor evolution and the emergence of resistant subclones (Table 1).

#### 5.1.7. Predictive Modeling and Disease Diagnosis

AI-driven predictive modeling is revolutionizing disease diagnosis and prognosis by leveraging large-scale genomic and clinical datasets. In particular, these models have an enormous value for identifying risk, characterizing tumor biology, predicting disease progression and personalizing treatment. By learning complex patterns in multi-dimensional data, classical machine learning (ML) and deep learning (DL) algorithms can go beyond conventional statistical tools to reveal novel insights into prognosis and disease diagnosis predictions (Figure 1).

#### 5.1.8. Disease Risk Prediction

AI models are increasingly used to predict an individual’s risk of developing diseases, particularly in genomics and cancer research. Supervised learning approaches employ known outcomes, such as disease status, to train models that map genetic or multi-omics patterns to risk. Classical algorithms include logistic regression and support vector machines (SVMs), which are useful for their interpretability and effectiveness in high-dimensional genomic data [155,156]. Decision trees and ensemble methods, including random forests and gradient boosting machines (XG-Boost), capture non-linear behavior between genetic variants and disease phenotypes while reducing overfitting, making them suitable for clinical prediction [157,158] (Table 1).

Recently, deep learning tools have been used to integrate germline variants, somatic mutations, transcriptomic profiles and clinical covariates, learning hierarchical attributes that improve prediction of complex characteristics such as cancer susceptibility [159]. For example, DeepRisk, a genome-wide deep neural network, outperformed classical PRS methods in Alzheimer’s disease, inflammatory bowel disease, type 2 diabetes and breast cancer [160] (Table 1).

Several studies show the translational potential of these supervised tools. Polygenic risk scores (PRS), often calculated using logistic regression or penalized regression such as LASSO, have been applied to stratify populations by breast and prostate cancer risk [161,162]. For non-mucinous ovarian cancer, penalized logistic regression using LASSO yielded a PRS that significantly stratified risk across ancestries [163]. Ensemble models such as random forests have identified multi-locus interactions associated with colorectal cancer susceptibility [164]. These examples highlight how supervised AI models can transform high-dimensional genomic data into practical insights for disease prediction, patient stratification and clinical decisions (Table 1).

#### 5.1.9. Precision Medicine

AI plays a crucial role in the development of precision medicine by analyzing genomic data to identify potential therapeutic targets and predict patient responses to treatments. The employment of machine learning algorithms could help to stratify patients based on their genetic profiles. This approach could improve the personalized treatment plans and, in turn, increase therapeutic efficacy and reduce adverse effects.

The identification of biomarkers, both gene expression patterns and mutation signatures, could be one of the main applications of AI tools. Those biomarker candidates could provide valuable information about tumor molecular pathways and possible therapeutic targets. On the one hand, supervised learning techniques, like random forests or support vector machines (SVMs), are frequently used to identify molecular patterns [157,158]. These studies allow us to distinguish between tumor and normal samples and identify biomarkers for early detection or prognosis of cancers. On the other hand, unsupervised learning methods, such as clustering, t-SNE and UMAP, could help to discover hidden patterns within omics data that may suggest the presence of unidentified molecular subtypes in heterogeneous cancer populations [165]. MOFA+ and single-cell VAE applications (scVI, totalVI) extract latent features that correlate with outcomes, while network integration methods such as Similarity Network Fusion reveal patient subgroups with different molecular phenotypes [149,154]. These tools have been applied to bulk and single-cell datasets to obtain candidate biomarkers and molecular subtypes for follow-up validation (Table 1).

In the case of patient stratification and clinical decision support, AI models can integrate multi-omics data, including genomics, epigenomics, transcriptomics and proteomics. In turn, they could classify patients into molecular subtypes, facilitating the identification of individuals most likely to benefit from specific therapies such as targeted inhibitors or immunotherapies. An example of that would be the use of deep learning algorithms, such as DeepGene and AutoPrognosis [166,167]. These AI tools have been used to integrate multi-omics data for comprehensive patient classification and to provide real-time decision support in clinical trials and precision oncology. Moreover, they can associate patients with individualized treatments based on their molecular profiles and improve treatment efficacy and patient outcomes (Table 1).

AI models could also be employed to predict drug response, especially to targeted therapies based on molecular characteristics, such as somatic mutations, copy number variations and gene expression profiles. By using pharmacogenomics datasets, such as the Cancer Cell Line Encyclopedia (CCLE) [168] and the Genomics of Drug Sensitivity in Cancer (GDSC) [169], and deep learning techniques, we could predict drug efficacy and identify biomarkers of drug resistance. Practical implementations include deep generative models that clearly model drug perturbation (Dr.VAE) and have demonstrated improved prediction of cell-line drug sensitivity [150] (Table 1). Additionally, AI plays an essential role in predicting drug resistance by analyzing genetic and transcriptomic data to identify mutations associated with resistance to specific therapies. This advance can help clinicians to anticipate the appearance of drug resistance and adjust therapeutic plans accordingly.

To model the evolutionary dynamics of tumors and predict how they will evolve over time under different therapeutic conditions, AI tools could be used. The integration of genetic, phenotypic and environmental data with tumor progression and response to therapies would improve the prediction accuracy of cancer evolution. The circulating tumor DNA (ctDNA) could be one of the key targets to investigate in-depth to detect minimal residual disease or early relapse signals [3]. Tools such as genome-wide fragmentomic classifiers (DELFI) are examples of how molecular time-series can precede radiologic progression and help anticipate emerging resistance [170] (Table 1).

## 6. Integrative Genomics and Multi-Omics Analysis

**Figure 2:**
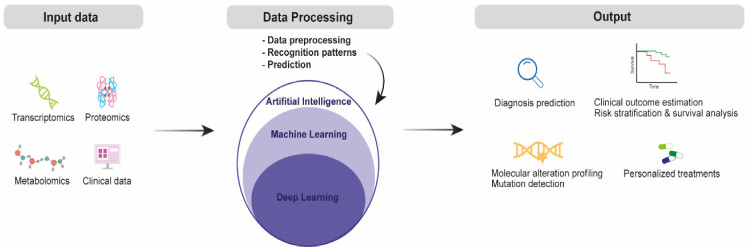
AI-Driven workflow for integrating omics and clinical data. The diagram shows how the different omics and clinical datasets are processed through AI-based methods, including preprocessing, feature extraction, and predictive modeling, to produce applicable biological and clinical insights.

The integration of multiple types of omics data, including genomic, transcriptomic, proteomic, epigenomic and metabolomic, allows researchers to gain a comprehensive understanding of the molecular mechanisms underlying health and disease. AI plays a pivotal role in analyzing and integrating these complex datasets, enabling the discovery of novel information that might be lost when examining each data type independently (Figure 2).

### 6.1. Multi-Omics Data Integration

The integration of multi-omics data is an essential process to combine data from different biological origins and build a cohesive model reflecting the interactions and regulatory mechanisms at play. AI techniques, particularly machine learning and deep learning, are tools for achieving this integration.

Multi-modal learning in cancer research has made a significant step by facilitating models to simultaneously combine diverse omics data, such as gene expression, DNA methylation, miRNA and copy number alterations, alongside clinical data. This approach has improved the prediction of disease states and outcomes. For example, the DeepMoIC algorithm employs a Graph Convolutional Network (GCN) architecture to integrate mRNA expression, miRNA and methylation data to classify cancer subtypes with high accuracy, overcoming models trained on single-omics alone [171]. Similarly, the MOLUNGN model integrates similar data and uses a combination of Graph Attention Networks and omics-specific modules followed by a correlation discovery fusion layer, allowing excellent stage classification in non-small cell lung cancer and discovering specific biomarkers [172] (Table 1).

Tensor and matrix factorization are some learning techniques of data fusion and representation. A recent study employs deep embedding and fusion of multiple omics types to improve prediction of drug response compared to single-omic models [173]. Additionally, the MOGAT tool uses matrix- or attention-based fusion to combine omics datasets and demonstrates that the fused model provides better prognostic stratification in breast cancer than baseline models [174] (Table 1).

Network-based approaches, particularly Graph Neural Networks, have become increasingly powerful for modeling complex molecular interactions. The Geometric graph neural network (GGNN) for multi-omics survival prediction in multiple myeloma and TCGA cancer types includes geometric characteristics from protein–protein interaction networks and pathway information to improve interpretability and accuracy of survival predictions [175]. Another example is the comparative analysis of multi-omics integration using different GNN architectures (GCN, GAT, GTN) applied to mRNA, miRNA and methylation data across 31 cancer types, which showed that attention-based GNNs (GAT) often offer better performance, especially when graph structure and biological prior knowledge are well integrated [8] (Table 1).

### 6.2. Functional Genomics

AI has accelerated functional genomics by converting raw sequence and epigenomic data into mechanistic hypotheses about how variants and regulatory elements affect gene function, cell states and disease. Modern approaches are classified into three, partly overlapping families: variant-effect prediction for coding and non-coding changes, identification and interpretation of regulatory elements from epigenomic and sequence data and pathway and network modeling that integrates multi-omic evidence to prioritize drivers and vulnerable nodes for intervention.

Deep learning has substantially improved the ability to predict variant effects, both coding and non-coding variants. DeepMind AlphaFold showed that accurate protein structure prediction from sequence is feasible at the proteome scale, creating a base to infer how missense variants might perturb folding or interactions [176]. Recently, protein language models have been used to score the impact of missense variants across the proteome, providing genome-wide effect estimates for millions of possible substitutions [177]. For identifying genetic variants, tools like DeepVariant use deep learning to transform raw DNA sequencing data into highly accurate variant calls [178]. In the case of non-coding variant predictions, which act through regulatory mechanisms, there are models such as DeepSEA and convolutional or attention architectures like Basenji and Enformer [179,180]. These tools learn the regulatory code directly from large chromatin-profiling datasets and can predict effects of single-nucleotide changes on chromatin marks and gene expression across cell types (Table 1). These models capture long-range interactions and cell-type specificity, improving in silico perturbation screens that prioritize candidate regulatory variants for experimental follow-up.

On the other hand, AI could also improve regulatory element discovery by combining epigenomic assays (ATAC-seq, DNase-seq, ChIP-seq) with sequence context and 3D contacts to nominate enhancers, promoters and insulators. These deep models trained increase sensitivity and resolution relative to peak-calling alone, and combined with experimental perturbations, they help to establish causal links between elements and target genes [180,181].

Pathways and network analysis powered by AI have the aim to discover pathways, modules and regulatory hubs that drive disease. Network-aware methods, which range from probabilistic graph models to graph neural networks, use protein–protein interaction maps, co-expression networks and mutation co-occurrence to highlight convergent pathway perturbations across patients and tumor types. Great pan-cancer efforts have shown that integrating non-coding and coding alterations into pathway models uncovers recurrently disrupted biological processes that single-variant analyses miss [182,183].

**Table 1:** Comprehensive overview of AI tools and techniques used in genomic and cancer research.

Name	Type	Applicability	Environment	Input Data Type	Output/Clinical Metric	Key Performance Metric	Availability	Ref.
***FastQC***	Rule-based QC tool	Quality Control	R (fastqcr)	Raw FASTQ reads	Per-base quality scores, adapter content and duplication rate	Pass/Warn/Fail per module; Phred score distribution	Open source/GUI + CLI	[133]
***MultiQC***	Aggregation/QC summarization tool	Quality Control	Python	QC reports (FastQC, STAR, etc.)	Aggregated HTML report with cross-sample QC metrics	Visual summary: no standalone accuracy metric	Open source/CLI	[133]
***Trimmomatic***	Rule-based preprocessing tool	Quality Control	Java	Raw FASTQ reads	Trimmed reads; adapter-cleaned sequences	Read survival rate, adapter removal efficiency	Open source/CLI	[134]
***SeqQscorer***	Machine-learning QC tool	Quality Control	Python (GitHub)	FASTQ/aligned reads	Binary QC pass/fail classification	AUC-ROC; accuracy on curated QC labels	Public repository GitHub	[135]
***MAC-ErrorReads***	AI-based error detection	Quality Control	Python	Sequencing reads (NGS)	Flagged error-containing reads	Precision/Recall for error detection	Public repository GitHub	[136]
***DeepDNA***	Deep learning (CNN-LSTM hybrid)	Data Compression	Python (GitHub)	Raw DNA sequences	Compressed binary representation	Compression ratio vs. GZip/reference methods	Public repository GitHub	[138]
***GeCo3***	Statistical/DNA compression algorithm	Data Compression	Python	DNA/RNA sequences (FASTA)	Compressed genomic files	Bits-per-symbol (bps); compression ratio	Open source/CLI	[139]
***ctGAN***	Generative Adversarial Network	Data Augmentation	Python	Tabular genomic/clinical data	Synthetic patient records/gene expression matrices	FID; statistical similarity (Wasserstein distance)	Open source	[5]
***scMMGAN***	Multi-modal GAN	Data Augmentation	Python	Single-cell multi-omics (RNA + ATAC)	Augmented cross-modal single-cell profiles	MMD; downstream clustering accuracy	Open source	[1]
***scMASKGAN***	Masked GAN	Data Augmentation	Python	scRNA-seq count matrices	Imputed/augmented gene expression data	RMSE on masked genes; cell-type preservation	Open source	[140]
***LSH-GAN***	GAN with locality-sensitive hashing	Data Augmentation	Python	High-dimensional genomic vectors	Synthetic genomic samples	Sample diversity score; classifier performance uplift	Open source	[141]
***DeepVariant***	Convolutional Neural Network (CNN)	Variant Calling	Python	Aligned BAM + reference genome	SNP/indel VCF calls	F1-score; precision/recall vs. GATK (Illumina WGS)	Open source/Cloud	[142]
***DeepSom***	CNN model	Variant Calling	Python	Tumor/normal paired BAM	Somatic SNV/indel VCF	Sensitivity/specificity for somatic mutations	Open source	[7]
***NeuSomatic***	CNN model	Variant Calling	Python	Tumor/normal BAM (WGS/WES)	Somatic variant VCF	F1-score; comparison vs. Mutect2/VarScan2	Open source	[143]
***VarNet***	End-to-end CNN architecture	Variant Calling	Python	Raw sequencing pileups	SNP/indel VCF with confidence scores	AUC; F1-score on benchmark datasets (e.g., GIAB)	Open source	[144]
***DeNovoCNN***	CNN model	Variant Calling	Python	Parent-offspring trio BAM files	De novo mutation calls (VCF)	Precision/recall for de novo variants	Open source	[6]
***EvoLSTM***	Recurrent Neural Network (LSTM)	Variant Calling/Mutation Modeling	Python	DNA sequences; evolutionary alignments	Mutation probability scores per position	Perplexity; correlation with observed mutation rates	Open source	[145]
***Lokatt***	Residual Neural Network + LSTM	Variant Calling (Nanopore basecalling)	Python (GitHub)	Nanopore raw signal (fast5/pod5)	Base-called FASTQ + variant calls	Basecalling accuracy; SNP F1-score (Nanopore data)	Public repository GitHub	[146]
***DAVI***	RNN/LSTM model	Variant Calling	Python	Aligned reads (BAM/pileup)	Indel VCF calls	Precision/recall for indels; comparison vs. GATK	Open source	[147]
***Dr.VAE***	Variational Autoencoder (VAE)	Gene Expression Analysis	Python	Bulk RNA-seq/drug response matrices	Latent embeddings; drug-response predictions	Pearson r; RMSE vs. observed IC50/EC50	Open source	[150]
***ScVI***	Deep generative model (VAE)	Gene Expression Analysis	Python (scvi-tools)	scRNA-seq count matrices (10x, Smart-seq)	Normalized expression; batch-corrected latent space	UMAP cluster purity; ASW; kBET batch correction score	Open source	[149]
***TotalVI***	VAE for multimodal single-cell data	Gene Expression Analysis	Python (scvi-tools)	CITE-seq (RNA + protein)	Denoised RNA + protein; integrated latent space	Pearson r for protein imputation; batch mixing entropy	Open source	[149]
***MOFA+***	Factor analysis/Probabilistic model	Gene Expression Analysis	R (MOFA2)	Multi-omics matrices (RNA, methylation, ATAC)	Latent factors explaining multi-omic variance	% variance explained per factor; enrichment analysis	Open source	[154]
***LASSO***	Penalized Regression	Disease Risk Prediction	R/Python (scikit-learn)	Genomic (SNP array/PGS) + clinical features	Disease risk score (continuous or binary)	AUC-ROC; C-statistic; calibration curve	Open source/CRAN + PyPI	[163]
***Support Vector Machines (SVM)***	Classical ML	Disease Risk Prediction	R/Python (scikit-learn)	Genomic + clinical tabular data	Binary or multi-class disease classification	AUC-ROC; accuracy; F1-score	Open source/CRAN + PyPI	[156]
***Random Forests***	Ensemble Model	Disease Risk Prediction	R/Python (scikit-learn)	Genomic + clinical tabular data	Risk class label + feature importance scores	AUC-ROC; OOB error; SHAP values	Open source/CRAN + PyPI	[157]
***XGBoost***	Gradient Boosting	Disease Risk Prediction	R/Python (xgboost)	Genomic + clinical tabular data	Risk score/class label	AUC-ROC; log-loss; SHAP feature importance	Open source/CRAN + PyPI	[158]
***DeepRisk***	Deep Learning	Disease Risk Prediction	Python	Multi-omics + EHR data	Polygenic/composite disease risk score	AUC-ROC; comparison vs. traditional PRS methods	Open source	[160]
***Autoencoders***	Deep Learning	Disease Risk Prediction	Python (TF/PyTorch)/R (keras)	High-dimensional omics data (RNA-seq, methylation)	Low-dimensional risk embeddings; anomaly scores	Reconstruction loss; downstream AUC-ROC	Open source	[148]
***Graph Neural Networks (GNNs)***	Deep Learning	Disease Risk Prediction	Python	PPI/gene regulatory networks + omics	Node-level or graph-level risk predictions	AUC-ROC; accuracy on pathway-based benchmarks	Open source	[175]
***DeepGene***	Deep learning classifier	Precision Medicine	Python	Gene expression profiles (RNA-seq)	Cancer subtype/treatment-response class	Accuracy; F1-score across cancer subtypes (TCGA)	Open source	[167]
***AutoPrognosis***	Automated ML framework	Precision Medicine	Python (autoprognosis)	Clinical + omics tabular data	Survival/prognostic risk score	C-index (Harrell); time-dependent AUC; Brier score	Open source/PyPI	[166]
***DELFI***	Fragmentomics/Machine learning	Precision Medicine	Python (GitHub)	Cell-free DNA (cfDNA) fragmentomics (WGS)	Cancer detection score; tissue-of-origin prediction	AUC-ROC; sensitivity at 98% specificity (liquid biopsy)	Public repository GitHub	[170]
***DeepMoIC***	Graph deep learning model	Multiomic Integration	Python	Multi-omics graphs (RNA, CNV, methylation)	Patient subtype clusters; survival risk groups	C-index; clustering NMI; comparison vs. MOFA/SNF	Open source	[171]
***MOLUNGN***	Graph neural network	Multiomic Integration	Python (GitHub)	Multi-omics patient graphs (lung cancer)	Survival prediction; molecular subtype labels	C-index; log-rank *p*-value for survival stratification	Public repository GitHub	[172]
***MOGAT***	Multi-omics Graph Attention Network	Multiomic Integration	Python	Multi-omics (RNA-seq, miRNA, methylation)	Disease classification scores; attention-weighted features	AUC-ROC; accuracy across TCGA cancer types	Open source	[174]
***DeepSEA***	CNN for regulatory genomics	Functional Genomics	Python (TensorFlow)	DNA sequence (±1 kb windows around variants)	Chromatin effect scores for variants (transcription factor binding, DNase, histone marks)	AUC-ROC for chromatin features; eQTL enrichment	Open source	[179]
***Basenji***	CNN for sequence-to-signal modeling	Functional Genomics	Python	DNA sequence (large genomic windows, ~128 kb)	Predicted regulatory signal tracks (ATAC, RNA-seq)	Pearson r vs. experimental signal; variant effect scores	Open source	[179]
***Enformer***	Transformer-based deep model	Functional Genomics	Python (TensorFlow)	DNA sequence (~196 kb windows)	Gene expression + epigenetic track predictions; variant effect scores	Pearson r; comparison vs. Basenji on Roadmap Epigenomics	Open source	[181]

Abbreviations: QC = Quality Control; CNN = Convolutional Neural Network; LSTM = Long Short-Term Memory; GAN = Generative Adversarial Network; VAE = Variational Autoencoder; GNN = Graph Neural Network; WGS = Whole Genome Sequencing; WES = Whole Exome Sequencing; scRNA-seq = single-cell RNA sequencing; AUC-ROC = Area Under the Receiver Operating Characteristic Curve; RMSE = Root Mean Square Error; NMI = Normalized Mutual Information; TCGA = The Cancer Genome Atlas; GIAB = Genome in a Bottle; cfDNA = cell-free DNA; PRS = Polygenic Risk Score; EHR = Electronic Health Record.

## 7. AI in Imaging Analysis

Artificial intelligence is transforming the field of medical imaging by helping the extraction of rich phenotypic and spatial information from MRI (magnetic resonance imaging), CT (computed tomography), PET (positron emission tomography), ultrasound and hybrid scans. These modalities generate high-dimensional data, often across time (longitudinal), which challenges manual analysis. Machine Learning and Deep Learning algorithms are now used not only for diagnosis, but also for tumor characterization, monitoring evolution and predicting responses over time (Figure 2).

### 7.1. Enhancing Image Acquisition and Quality

Image reconstruction and noise reduction methods based on deep learning facilitate the production of high-resolution MRI and CT images faster or at lower doses than conventional tools. AI has allowed for an increase in the fidelity, speed and usability of imaging data. For example, an AI algorithm trained for ultra-low-dose CT in an emergency setting detected 5.8× more pulmonary nodules, including small and hard-to-see ones, while increasing false positives, showing both promises and disadvantages [184]. Moreover, AI detection of pulmonary ground-glass nodules on spectral detector CT (SDCT) using virtual monochromatic images exhibits more sensitivity for early change detection than conventional CT reconstructions [185].

### 7.2. Automated Image Segmentation

The accurate segmentation of tumor and anatomical structures is an important process to delimit the tumor area correctly and, in turn, avoid recurrence and metastasis. Convolutional neural networks (CNNs) and related architectures are used extensively. In head and neck cancer, longitudinal segmentation of tumor volumes using SegResNet with deep supervision and mask attention across pre- and mid- radiotherapy MRI scans gave high segmentation overlap (Dice similarity coefficient, DSC) and improved consistency compared to single-timepoint models [186]. Another study on breast cancer (BIRADS Category 4 lesions via DCE-MRI) used AI-based automatic segmentation to predict lesion nature. The AI-radiomics model was better than manual segmentation approaches in terms of both accuracy and consistency compared to radiologists’ results [187].

### 7.3. Tumor Characterization and Classification

After segmentation, AI models can facilitate the quantification of image features (radiomics), the characterization of tumor biology and the classification of tumor types. For instance, in a multicentre study, it integrated longitudinal MRI spatial habitat radiomics, transcriptomics and single-cell RNA sequencing in breast cancer. This study allowed the development of a multi-modality model to predict pathological complete response to neoadjuvant therapy with high AUCs in external validation and correlated with immune responses, such as B cell infiltration [188]. Furthermore, the employment of Deep learning in sinonasal malignancies has allowed for the differentiation of different histologies, such as squamous cell carcinoma and adenocarcinoma, with high accuracy [189]. In addition, advances in brain tumor analysis have demonstrated that hyperspectral imaging combined with machine-learning techniques can accurately identify and delineate both primary and secondary brain tumors in vivo, offering a promising intraoperative decision-support tool for real-time tumor boundary detection and improved surgical precision [190].

### 7.4. Monitoring Tumor Evolution and Treatment Response

To understand how tumors evolve under treatment requires an evaluation by imaging over time. AI models using longitudinal imaging detect imperceptible changes that may precede clinical or radiologic evidence of progression. In hepatocellular carcinoma, a longitudinal CE-MRI-based Siamese network model was used to predict response to drug-eluting bead transarterial chemoembolization (DEB-TACE), showing that combining temporal imaging changes with DL improves prediction of response [191].

### 7.5. Early Detection, Accuracy and Efficiency Gains

AI tools are allowing for the improvement of the early detection and the diagnosis accuracy in some fields of cancer research. In lung cancer, AI assistance of chest CT scans significantly increased sensitivity to detect nodules, including ones that radiologists missed [192]. Automated matching of pulmonary nodules on follow-up CT also helps maintain diagnostic continuity and detect changes over time [193]. Multi-modal radiomics combining ultrasound and MRI has been used to predict disease-free survival in breast cancer, showing better prognosis prediction than single-modality models [194].

## 8. Mathematical Modeling, Computer Simulations and AI in Tumor Growth Dynamics

Mathematical modeling and computational simulation have become increasingly important for understanding tumor growth dynamics, clonal evolution and therapeutic adaptation. Long before the current expansion of artificial intelligence (AI) in oncology, mathematical oncology attempted to describe cancer progression using systems of differential equations, stochastic processes, agent-based models, cellular automata and spatial ecological simulations. These approaches provide mechanistic representations of tumor behavior that complement purely data-driven AI methodologies.

Early mathematical models of tumor growth relied on deterministic equations such as exponential, logistic and Gompertzian growth laws to describe tumor expansion kinetics [195,196]. Although these models captured basic proliferative behavior, they were limited in representing intratumoral heterogeneity, spatial organization and evolutionary adaptation. More recent computational approaches incorporate stochasticity, spatial constraints, microenvironmental interactions and clonal selection, thereby providing more biologically realistic descriptions of tumor evolution [197,198].

Agent-based models (ABMs) and cellular automata have been particularly useful for simulating tumor heterogeneity and microenvironmental adaptation. In these systems, individual cancer cells behave as autonomous agents capable of proliferation, migration, mutation, cooperation and competition according to predefined biological rules [199,200]. These models reproduce emergent properties observed in real tumors, including invasive fronts, hypoxic niches, clonal sweeps and therapy-resistant populations. Importantly, such simulations demonstrate that complex tumor behaviors can arise from relatively simple local interactions between cells and their environment, reinforcing the concept of tumors as adaptive self-organizing systems.

Recent developments increasingly combine mathematical oncology with machine learning and AI. Hybrid AI-mechanistic models integrate longitudinal genomic, imaging and clinical datasets with evolutionary simulations to improve prediction of tumor progression and therapeutic response [201,202]. Deep learning approaches can infer hidden parameters from experimental and clinical data, while mechanistic models provide biologically interpretable constraints that improve robustness and generalizability. This integration is particularly relevant for predicting resistance evolution under therapeutic pressure, where purely statistical AI models frequently fail to capture causal evolutionary dynamics.

Reinforcement learning and generative AI models are also being explored to simulate tumor adaptation under different treatment schedules and to optimize adaptive therapy strategies [203,204]. In this context, AI functions not only as a predictive tool but also as a computational framework capable of testing virtual therapeutic interventions in silico before clinical implementation. Such approaches are closely related to the emerging concept of “digital twins” in oncology, where continuously updated computational representations of individual tumors may eventually guide personalized evolutionary therapy [205].

Importantly, mathematical simulations have contributed to a conceptual shift in cancer biology. Tumor growth is no longer viewed solely as uncontrolled proliferation but rather as a dynamic evolutionary process governed by ecological interactions, adaptation and non-linear system behavior. AI-enhanced computational oncology therefore represents a convergence between systems biology, mathematical modeling, and evolutionary theory, providing a more mechanistic understanding of tumor evolution and resistance.

## 9. Evolutionary Game Theory, Artificial Life, Complexity Theory and Tumors as Self-Adaptive Intelligent Systems

Increasing evidence suggests that tumor evolution cannot be fully understood through reductionist genetic models alone. Cancer progression emerges from dynamic interactions among heterogeneous cell populations, the tumor microenvironment, immune surveillance and therapeutic pressures. Consequently, several theoretical frameworks derived from Evolutionary Game Theory (EGT), Artificial Life (ALife), Complexity Theory and self-organizing systems have been increasingly applied to oncology in order to model tumor adaptation, resistance and survival strategies.

Evolutionary Game Theory provides a mathematical framework for describing competition and cooperation among tumor cell populations under selective pressure [206,207]. In these models, cancer cells adopt different “strategies” associated with proliferation, metabolic adaptation, immune evasion, invasion or therapy resistance. Cellular fitness depends not only on intrinsic properties but also on interactions with neighboring cells and environmental conditions. EGT models have demonstrated how resistant populations may emerge through frequency-dependent selection, competitive release and ecological cooperation between subclones [208,209]. Importantly, these studies support adaptive therapy approaches aimed at controlling tumor ecosystems rather than attempting complete eradication.

Artificial Life and agent-based simulations extend these concepts by modeling tumors as decentralized adaptive systems composed of interacting autonomous entities [210]. Such systems frequently exhibit emergent behaviors including collective migration, metabolic cooperation, phenotypic plasticity and self-organization without central control. These properties resemble behaviors observed in other complex adaptive systems found in ecology, swarm intelligence and evolutionary biology. In this context, tumors can be interpreted as evolving ecosystems capable of information processing and dynamic adaptation.

Complexity Theory further supports this perspective by emphasizing non-linearity, feedback loops, stochastic fluctuations and emergent organization within tumor ecosystems [197]. Tumor progression is increasingly recognized as a process governed by multi-scale interactions spanning molecular, cellular, tissue and systemic levels. Small perturbations in microenvironmental conditions or therapeutic pressures may generate disproportionate evolutionary consequences, contributing to unpredictable resistance trajectories and phenotypic diversification.

Importantly, these theoretical approaches have contributed to a major conceptual shift in oncology: the possibility of considering tumors themselves as forms of adaptive intelligent systems. Although tumors do not possess cognition in the classical sense, they exhibit collective properties commonly associated with intelligent behavior, including self-organization, environmental sensing, adaptation, memory-like responses, distributed decision-making and survival optimization [211,212,213]. Tumor cell populations continuously process environmental information and dynamically adjust proliferative, metabolic and invasive programs to maximize survival under hostile conditions such as hypoxia, immune attack, and therapeutic intervention.

This perspective has important implications for the future development of AI in oncology. Current AI applications frequently treat tumors as passive datasets characterized by static molecular features. However, if tumors behave as adaptive evolutionary systems capable of collective survival strategies, AI models must incorporate ecological dynamics, evolutionary feedback, temporal adaptation and emergent behavior rather than relying exclusively on static predictive correlations. Integrating AI with Evolutionary Game Theory, ecological modeling and complexity-based frameworks may therefore improve the prediction of resistance emergence, metastatic dissemination and therapy-induced adaptation.

Ultimately, these approaches suggest that future oncology may require a transition from mutation-centered models toward systems-level evolutionary intelligence frameworks. In such a paradigm, AI would not simply classify tumors, but rather interact with continuously evolving biological systems characterized by adaptation, competition, cooperation and emergent collective behavior.

## 10. How AI Is Transforming the Biological Understanding of Tumor Evolution

### 10.1. AI as a Mechanistic Framework for Evolutionary Oncology

Traditional cancer biology has largely interpreted tumor progression through static molecular snapshots derived from bulk genomic or histopathological analyses [103,214]. While these approaches have substantially advanced the understanding of oncogenic mutations and signaling pathways, they frequently fail to capture the dynamic and adaptive nature of tumor evolution. Cancer is increasingly recognized as a complex evolutionary ecosystem characterized by continuous genetic diversification, phenotypic plasticity, environmental selection pressure, and therapy-driven adaptation [14,215,216]. In this context, artificial intelligence (AI) is emerging not merely as a computational tool for data analysis but as a transformative setting for reconstructing the biological mechanisms underlying tumor evolution. In parallel, mathematical oncology has developed mechanistic frameworks based on ordinary and partial differential equations to model tumor proliferation, invasion, angiogenesis and spatial diffusion, as well as stochastic and agent-based simulations to reproduce clonal evolution, tumor microenvironment interactions and phenotypic adaptation under selective pressure [217,218]. These models provide an essential theoretical basis for understanding tumor progression as a dynamic and spatially heterogeneous system. However, their predictive performance is frequently limited by parameter uncertainty and the difficulty of integrating high-dimensional biological data. In this context, AI approaches can complement mechanistic modeling through data-driven parameter inference, pattern recognition and optimization of tumor growth and evolutionary simulations [219].

Recent advances in AI-driven multi-modal integration have enabled simultaneous interrogation of genomic, transcriptomic, epigenomic, proteomic, metabolomic, spatial, and imaging datasets [220,221]. Unlike traditional analytical methods that examine these data types independently, AI models can identify latent relationships between molecular alterations, cellular phenotypes, and microenvironmental dynamics across temporal and spatial scales. This capability is fundamentally reshaping current concepts of tumor evolution by revealing that cancer progression is not solely driven by linear mutational accumulation, but rather by adaptive interactions among heterogeneous cell populations exposed to fluctuating ecological and therapeutic pressures.

One of the most important conceptual contributions of AI to evolutionary oncology lies in its ability to model tumors as dynamic systems rather than static disease entities. Machine learning approaches, particularly deep learning and graph-based neural networks, have demonstrated remarkable capability in reconstructing clonal phylogenies and predicting evolutionary trajectories from longitudinal patient datasets [74,222,223]. These models can infer patterns of clonal competition, selective sweeps, and subclonal diversification that are difficult to detect through conventional statistical approaches. As a result, AI is enabling a shift from descriptive oncology toward predictive evolutionary biology.

### 10.2. AI and the Decoding of Intratumoral Heterogeneity

Intratumoral heterogeneity is a defining hallmark of tumor evolution and represents a major obstacle to effective therapy. Heterogeneous tumor cell populations differ in genomic composition, transcriptional state, metabolic activity, proliferative potential, and therapeutic sensitivity. Conventional bulk sequencing approaches often obscure these differences by averaging signals across millions of cells. AI-assisted single-cell and spatial transcriptomic analyses have significantly improved the ability to resolve these complex cellular ecosystems [224,225].

Deep learning algorithms can now identify previously unrecognized cellular subpopulations associated with metastasis, immune evasion, stemness, and drug resistance. Importantly, these approaches reveal that tumor heterogeneity is not simply stochastic noise but frequently reflects structured evolutionary adaptation to environmental constraints such as hypoxia, nutrient limitation, immune surveillance, and therapeutic exposure. AI-based clustering and trajectory inference methods have further demonstrated that cellular phenotypes exist along continuous transitional spectra rather than discrete states, thereby challenging traditional binary classifications of tumor cell identity.

**Figure 3:**
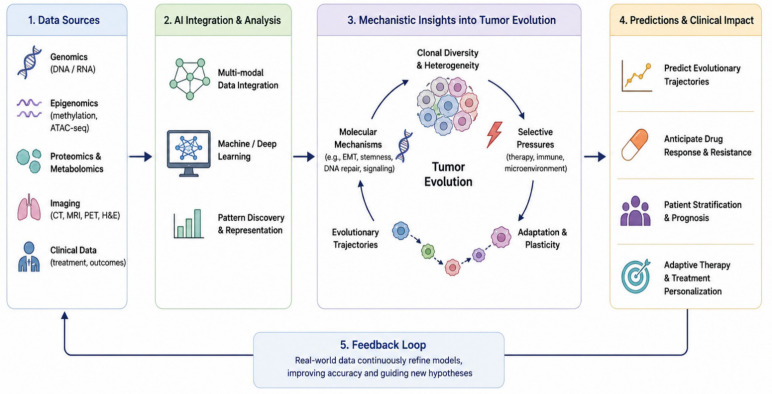
Artificial intelligence as a mechanistic framework for understanding tumor evolution. The figure summarizes the emerging role of artificial intelligence (AI) in evolutionary oncology. Multi-modal biological and clinical data, including genomics, epigenomics, proteomics, imaging, and clinical information, are integrated through machine learning and deep learning approaches. AI-based analyses identify hidden biological patterns associated with tumor evolution, including clonal heterogeneity, selective pressures, cellular plasticity, and adaptive evolutionary trajectories. These mechanistic insights enable prediction of tumor progression, therapeutic resistance, patient stratification, and adaptive treatment strategies. Continuous incorporation of real-world data further refines predictive models and supports the development of evolutionary precision oncology.

This has major implications for understanding epithelial–mesenchymal transition (EMT), cancer stemness, and lineage plasticity [226,227]. AI-driven analyses indicate that transitional intermediate states may possess enhanced metastatic and drug-resistant potential compared with fully differentiated phenotypes. Such findings support the emerging concept that tumor evolution is governed not only by genetic mutations but also by dynamic cell-state plasticity regulated by epigenetic and microenvironmental interactions (Figure 3). In this context, concepts from Evolutionary Game Theory and complexity science have been applied to model intratumoral heterogeneity as the result of competitive and cooperative interactions among distinct tumor subclones occupying heterogeneous ecological niches within the tumor microenvironment [197,228]. These frameworks suggest that phenotypic diversification and clonal coexistence may emerge through frequency-dependent interactions, spatial resource competition and adaptive fitness trade-offs among tumor cell populations.

### 10.3. AI Reveals Tumor Evolution as an Ecological Process

Another major conceptual advance enabled by AI is the reinterpretation of tumors as evolving ecological systems. Tumor progression involves continuous interactions between malignant cells and surrounding stromal, vascular, and immune components within the tumor microenvironment (TME). These interactions generate selective pressures that shape clonal fitness and adaptive survival. AI-assisted spatial omics and imaging analyses have revealed highly organized spatial architectures within tumors, including immune-excluded niches, hypoxic regions, metabolic gradients, and stromal interaction networks [229,230]. Such spatially resolved analyses demonstrate that tumor evolution occurs within geographically heterogeneous microenvironments that promote distinct evolutionary trajectories.

Moreover, AI models have shown that immune editing represents a major evolutionary force driving tumor adaptation. Machine learning approaches integrating T-cell receptor repertoires, cytokine signaling, and neoantigen burden have identified mechanisms through which tumors dynamically evade immune recognition during progression and immunotherapy exposure [231]. These findings support the emerging paradigm that cancer evolution resembles an ecological arms race involving reciprocal adaptation between tumor cells and host immunity.

Importantly, AI-driven ecological modeling has also provided insight into therapy-induced evolutionary bottlenecks. Cytotoxic therapy, targeted therapy, and immunotherapy impose selective constraints that eliminate sensitive populations while enriching resistant clones or drug-tolerant persistent cells. AI models trained on longitudinal clinical data increasingly demonstrate the ability to predict these adaptive shifts before overt clinical relapse becomes detectable.

### 10.4. AI and the Mechanisms of Therapeutic Resistance

Therapeutic resistance represents one of the clearest manifestations of tumor evolution. Historically, resistance was often interpreted as the consequence of isolated genetic mutations conferring drug insensitivity. However, AI-driven integrative analyses suggest that resistance is a multifactorial evolutionary process involving genomic instability, transcriptional reprogramming, epigenetic remodeling, metabolic adaptation, and microenvironmental interactions.

Deep learning frames integrating multi-omics data have identified resistance-associated transcriptional programs linked to hypoxia signaling, oxidative stress responses, EMT activation, and immune suppression [232,233,234,235]. Furthermore, AI-based trajectory analyses indicate that resistant phenotypes frequently emerge from pre-existing rare subclonal populations or transient adaptive cell states rather than from entirely new mutational events.

This insight has important clinical implications. Rather than viewing resistance as an unavoidable endpoint, AI-supported evolutionary modeling suggests that resistance may be forecasted and potentially redirected through adaptive therapeutic strategies. Evolutionary game theory and reinforcement learning models are increasingly being applied to simulate tumor adaptation under different treatment schedules, thereby enabling optimization of therapeutic sequencing and dose modulation [236,237,238]. Complementary work in mathematical oncology and computer simulations of tumor growth further supports this predictive framework by enabling in silico exploration of treatment-induced evolutionary dynamics, including the emergence of drug-resistant subclones, therapy-driven selection pressures and optimal scheduling strategies such as adaptive therapy and dose modulation [239].

These developments are contributing to the emergence of adaptive oncology, in which treatment is dynamically adjusted according to predicted evolutionary responses rather than fixed protocol-based regimens (Figure 3).

### 10.5. Toward Predictive and Evolutionary Precision Oncology

The integration of AI into cancer biology is contributing to a paradigm shift from mutation-centered precision medicine toward evolutionary precision oncology. Conventional precision oncology primarily focuses on identifying actionable driver mutations at a single time point. In contrast, AI-enabled evolutionary oncology aims to predict how tumors will adapt over time under changing environmental and therapeutic conditions (Figure 3).

Future AI systems may integrate real-time molecular profiling, circulating tumor DNA monitoring, spatial imaging, and clinical parameters to construct continuously updated digital representations of tumor evolution [4,240]. These so-called “digital twins” could potentially simulate future evolutionary trajectories and guide individualized therapeutic interventions before resistant populations emerge.

Importantly, this recognizes cancer as a probabilistic and adaptive system rather than a static genetic disease. AI therefore provides not only computational scalability but also a conceptual bridge connecting systems biology, ecology, evolutionary theory, and clinical oncology.

## 11. Ethical Considerations

The fast integration of artificial intelligence (AI) into oncology raises deep ethical challenges that extend beyond other areas of medicine. Key concerns include equity, privacy, autonomy and the balance between human and machine judgment in patient care [241,242,243]. AI in oncology is remarkably complex because it involves two interrelated genomes, germline and somatic, which increases issues of bias and representation [242,244]. Given that there are overrepresentations of specific racial populations in cancer biobanks, algorithmic predictions may inadvertently reinforce the systemic and structural inequities in cancer care [245,246]. The use of AI in cancer research involves handling sensitive patient data, raising concerns about privacy and confidentiality. Ensuring robust data security measures and obtaining informed consent are crucial for maintaining patient trust and compliance with regulations.

Patient autonomy is a major issue because the AI models are optimized for outcomes such as overall survival, forgetting the individual patient preferences. This practice leads to care based on algorithm-driven rather than patient-centered [247,248]. In some studies, discrepancies were observed between AI-generated treatment recommendations and the oncologists’ decisions, sometimes because of differences in patients’ values [247]. For these reasons, it is essential to have transparency about how data are used, when patients consent to their use and how algorithms apply those data to care decisions [243].

On the other hand, the lack of normalized frameworks for divulging dataset composition and extrapolation processes increases the risk that biased or incomplete data will guide clinical decisions [242,244]. This problem is made worse by the blind nature of many AI model predictors, which reduce the trust between clinicians and patients [244,249]. Ensuring the interpretability of these models is essential for gaining insights into tumor evolution and making clinically relevant decisions.

Public enthusiasm for AI in oncology is strong, with high levels of trust in AI for cancer detection and survival prediction [250]. The oncologists have more caution, particularly in AI systems that move diagnostics into direct clinical decisions [241]. This skepticism reflects past experiences with overhyped medical innovations that do not give good results or cause harm [251,252]. Therefore, it is essential to safeguard patient privacy and safety to ensure trustworthy and ethically responsible cancer care.

## 12. Conclusions, Limitations and Future Directions

Artificial intelligence has changed the study of tumor evolution in cancer research. The use of AI models to analyze large amounts of multi-omics, imaging and clinical data has allowed researchers to raise novel knowledge about the mechanisms driving tumor heterogeneity, progression and resistance. These advances can improve the development of more effective and personalized treatments and, in consequence, enhance patient outcomes. AI can be employed to identify patient subgroups that are likely to benefit from experimental therapies, optimizing the design and execution of clinical trials. Moreover, those tools can enable real-time monitoring of tumor evolution during treatment, detecting early signs of resistance and getting individualized therapies. However, it is essential to address ethical and practical challenges for managing the full potential of AI in cancer research while preserving patient care and safety.

Over the next 5–10 years, AI is expected to transition from static pattern recognition toward evolutionary forecasting grounded in explicit fitness landscapes. Continuous integration of circulating tumor DNA, imaging, and clinical data may enable real-time adaptive oncology, while hybrid mechanistic–AI models could improve interpretability and robustness. Patient-specific digital tumor twins may allow in silico testing of therapeutic strategies prior to clinical deployment.

Despite these advances, several major challenges remain. Many AI models continue to function as “black boxes” with limited mechanistic interpretability, reducing biological transparency and clinical trust. Additionally, most available datasets remain cross-sectional rather than longitudinal, limiting the ability to accurately reconstruct temporal evolutionary dynamics. Tumor evolution is also influenced by stochastic events, environmental variability, and patient-specific biological factors that may not be fully captured by current computational settings.

Another critical limitation involves causality. While AI models excel at identifying correlations and predictive patterns, distinguishing causal drivers from associated features remains difficult. Integrating mechanistic biological experiments with AI-based inference will therefore be essential for validating the evolutionary hypotheses generated.

Ultimately, AI will not replace evolutionary theory in oncology; rather, its transformative potential lies in operationalizing evolutionary principles at clinical scale to enable predictive, adaptive, and equitable cancer care.

## Data Availability

No new data has been generated in this work.

## Abbreviations

The following abbreviations are used in this manuscript:

AI	Artificial intelligence
CAFs	cancer-associated fibroblasts
CCLE	Cancer Cell Line Encyclopedia
CML	chronic myeloid leukemia
CNN	Convolutional Neural Network
CSC	Cancer stem cells
CT	computed tomography
ctDNA	circulating tumor DNA
DL	deep learning
EMT	epithelial–mesenchymal transition
GANs	Generative Adversarial Networks
GAT	attention-based GNNs
GCNs	Graph Convolutional Networks
GDSC	Genomics of Drug Sensitivity in Cancer
GRU	Gated Recurrent Unit
HIFs	hypoxia-inducible factors
ITH	Intratumoral heterogeneity
LSTMs	Long Short-Term Memory
MDR	multidrug resistance
MDSCs	myeloid-derived suppressor cells
ML	Machine learning
MRI	magnetic resonance imaging
NGS	next-generation sequencing
NSCLC	non-small cell lung cancer
PET	positron emission tomography
PRS	Polygenic risk scores
RNNs	Recurrent Neural Networks
scMASKGAN	masked multi-scale CNN + attention-enhanced GAN
SNPs	single nucleotide polymorphisms
SVMs	support vector machines
TAMs	tumor-associated macrophages
TCGA	The Cancer Genome Atlas
TGF-β	transforming growth factor-beta
TME	tumor microenvironment
Tregs	regulatory T cells
VAEs	Variational autoencoders
VEGF	Vascular Endothelial Growth Factor
